# Structure Extension of Tree-Augmented Naive Bayes

**DOI:** 10.3390/e21080721

**Published:** 2019-07-25

**Authors:** Yuguang Long, Limin Wang, Minghui Sun

**Affiliations:** 1 College of Software, Jilin University, Changchun 130012, China; 2 Key Laboratory of Symbolic Computation and Knowledge Engineering of Ministry of Education, Jilin University, Changchun 130012, China; 3 College of Computer Science and Technology, Jilin University, Changchun 130012, China

**Keywords:** tree-augmented naive Bayes, Kullback–Leibler divergence, attribute independence assumption, probability distribution

## Abstract

Due to the simplicity and competitive classification performance of the naive Bayes (NB), researchers have proposed many approaches to improve NB by weakening its attribute independence assumption. Through the theoretical analysis of Kullback–Leibler divergence, the difference between NB and its variations lies in different orders of conditional mutual information represented by these augmenting edges in the tree-shaped network structure. In this paper, we propose to relax the independence assumption by further generalizing tree-augmented naive Bayes (TAN) from 1-dependence Bayesian network classifiers (BNC) to arbitrary *k*-dependence. Sub-models of TAN that are built to respectively represent specific conditional dependence relationships may “best match” the conditional probability distribution over the training data. Extensive experimental results reveal that the proposed algorithm achieves bias-variance trade-off and substantially better generalization performance than state-of-the-art classifiers such as logistic regression.

## 1. Introduction

Supervised classification is an important task in data-mining and pattern recognition [1]. It requires building a classifier that can map an unlabeled instance into a class label. Traditional approaches to classification problems include decision trees, logistic regression etc. More recently, Bayesian network classifiers (BNCs) have attracted more attention from researchers in terms of explicit, graphical, interpretable representation and competitive performance against state-of-the-art classifiers.

Among numerous BNCs, naive Bayes (NB) is an extremely simple and remarkably effective approach to classification [2]. It infers the conditional probability by assuming that the attributes are independent given the class label [3]. It follows logically that relaxing NB’s independence assumption is a feasible and effective approach to build more powerful BNCs [4,5]. Researchers proposed to extend NB from 0-dependence BNC to 1-dependence BNCs [6,7] (e.g., tree-augmented naive Bayes or TAN), and then to arbitrary *k*-dependence BNCs [8,9] (e.g., *k*-dependence Bayesian classifier or KDB). These BNCs learn from training data and allow additional edges between attributes that capture the dependence relationships among them. These restricted BNCs also capture another assumption behind NB, i.e., every attribute is dependent on the class variable and thus the class is the root in the network.

Given a random instance $x=(x_{1},\cdots ,x_{n})$, where $x_{i}\in \Omega_{X_{i}}$, classification is done by applying Bayes rule to predict the class label $y^{*}$ that corresponds to the highest posterior probability of the class variable, i.e., $y^{*}=\arg\max P\left(y|x\right)$, where $y\in \Omega_{y}$. By using Bayes theorem, for restricted BNC we have

(1) $$y^{*}=\arg\max P\left(y|x\right)=\arg\max\frac{P(y,x)}{P(x)}=\arg\max P\left(y,x\right)=\arg\max P\left(x|y\right)P\left(y\right)$$

The objective of restricted BNC learning is to induce a network (or a set of networks) that may “best match” the conditional probability distribution P(x|y) given different class labels over the training data and explicitly represent statements about conditional independence. Information theory, which is proposed by Shannon, has established mathematical basis for the rapid development of BN. Mutual information (MI) $I(X_{i};Y)$ is the most commonly used criterion to rank attributes for attribute sorting or filtering [10,11], and conditional mutual information (CMI) $I(X_{i};X_{j}|Y)$ is used to measure conditional dependence between attribute pair $X_{i}$ and $X_{j}$ for identifying possible dependencies.

Among numerous proposals to improve the accuracy of NB by weakening its attribute independence assumption, TAN demonstrates remarkable classification performance, yet at the same time maintains the computational simplicity and robustness that characterize NB. However, it can only model 1-dependence relationships among attributes. The optimization process of BNCs is implemented in practice by using heuristic search techniques to find the best candidate over the space of possible networks. The search process relies on a scoring function that evaluates each network with respect to the training data, and then to search for the best network according to this function. The likelihood function, e.g., Kullback–Leibler divergence, plays a fundamental role in Bayesian statistics [12,13]. The likelihood principle states that all relevant information for inference is contained in the likelihood function for the observed data given the assumed statistical model. We prove from the viewpoint of Kullback–Leibler divergence that the difference between NB and its variations lies in different orders of CMIs represented by these augmenting edges in the tree-shaped network structure. The CMIs may vary greatly for different class labels. Thus, in this paper we propose to generalize TAN from 1-dependence BNC to arbitrary *k*-dependence one. Different sub-models of TAN are introduced to respectively represent specific conditional dependence relationships depending on *y*. The Bayes rule is applied to select the maximum of the joint probability distribution P(y,x) for classification. Extensive experimental results reveal that the proposed algorithm, called Extensive TAN (ETAN), achieves competitive generalization performance and outperforms several state-of-the-art BNCs such as KDB while retaining excellent computational complexity.

## 2. Prior Work

A BNC is a graphical representation of the joint probability distribution P(y,x). It comprises two components. Firstly, a directed acyclic graph G=(U,V), where U=X∪Y. $X=\{X_{1},\cdots ,X_{n}\}$ and *Y* respectively represent the attributes and class variable. V represents the set of arcs or direct dependencies. Secondly, a set of parameters, which are usually conditional probability distributions for each attribute in U. Given a training data set D, the goal of learning a BNC is to find the Bayesian network B that best represents P(u) or P(y,x) and predicts the class label for an unlabeled instance by selecting $\arg\max_{y}P\left(y,x\right)$. According to the chain rule of joint probability, P(y,x) is calculated by

(2) $$P\left(u\right)=P\left(y,x\right)=P\left(y\right)P\left(x_{1}|y\right)P\left(x_{2}|x_{1},y\right)\cdots P\left(x_{n}|x_{1},\cdots ,x_{n-1},y\right),$$

For discrete probability distributions P(u) and Q(u), the Kullback–Leibler divergence (also called relative entropy) is a measure of distance between these two probability distributions and is defined to be [14]

(3) $$KL\left(P||Q\right)=\sum_{u}P\left(u\right)\log\frac{P(u)}{Q(u)}=H_{Q}\left(U\right)-H_{P}\left(U\right)$$

It is the expectation of the logarithmic difference between P(u) and Q(u), where the expectation is taken using P(u). In other words, it is also the difference between $H_{Q}\left(U\right)$ and $H_{P}\left(U\right)$. Suppose that B is a Bayesian network over U, $P_{B}\left(u\right)$ is the joint probability encoded in B, the Kullback–Leibler divergence between the expected P(u) in Equation (2) and $P_{B}\left(u\right)$ is

(4) $$KL\left(P||B\right)=\sum_{u}P\left(u\right)\log\frac{P(u)}{P_{B}\left(u\right)}=H_{B}\left(U\right)-H_{P}\left(U\right)$$

where $H_{B}\left(U\right)=-\sum_{u}P\left(u\right)\log P_{B}\left(u\right)$. The entropy function $H_{P}\left(U\right)$ is the optimal number of bits needed to store all possible combinations of attribute values of U. Thus, KL(P||B) can measure the difference between the information quantity carried by *D* and that encoded in B.

**Figure 1:**
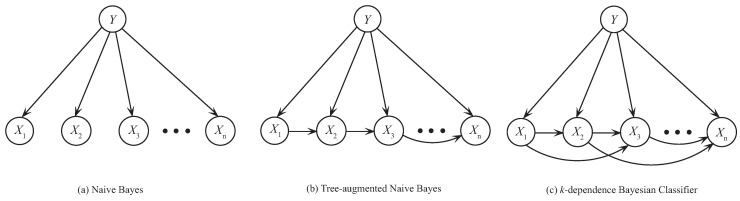
Examples of different BNCs. (**a**) Naive Bayes, (**b**) Tree-augmented naive Bayes, (**c**) *k*-dependence Bayesian Classifier.

NB, which is the simplest BNC, involves no dependence in its network structure according to conditional independence assumption [15]. Figure 1a shows an example of its network structure. Hence, for NB, $P_{NB}\left(u\right)$ is calculated by

(5) $$P_{NB}\left(u\right)=P\left(y\right)P\left(x_{1}|y\right)P\left(x_{2}|y\right)\cdots P\left(x_{n}|y\right),$$

Thus, $H_{NB}\left(U\right)$ can be calculated by

(6) $$\begin{array}{cc} H_{NB}\left(U\right) & =-\sum_{y,x_{1},\cdots ,x_{n}}P\left(y,x_{1},\cdots ,x_{n}\right)logP\left(y\right)P\left(x_{1}|y\right)P\left(x_{2}|y\right)\cdots P\left(x_{n}|y\right)\\ & =-\sum_{y,x_{1},\cdots ,x_{n}}P\left(y,x_{1},\cdots ,x_{n}\right)logP\left(y\right)-\sum_{y,x_{1},\cdots ,x_{n}}P\left(y,x_{1},\cdots ,x_{n}\right)\left\{\sum_{i=1}^{n}logP\left(x_{i}|y\right)\right\}\\ & =-\sum_{y}P\left(y\right)logP\left(y\right)-\sum_{i=1}^{n}\sum_{y,x_{1},\cdots ,x_{n}}\left\{P\left(y,x_{1},\cdots ,x_{n}\right)logP\left(x_{i}|y\right)\right\}\\ & =-\sum_{y}P\left(y\right)logP\left(y\right)-\sum_{i=1}^{n}\sum_{y,x_{i}}\left\{P\left(y,x_{i}\right)logP\left(x_{i}|y\right)\right\}\\ & =H\left(Y\right)+\sum_{i=1}^{n}H\left(X_{i}|Y\right) \end{array}$$

The remarkable classification performance of NB has stimulated the exploration of improving its classification performance [16]. However, the dependency relationships between attributes always violate this assumption in many learning tasks. Many methods [17,18,19,20] attempt to improve the classification performance of NB by relaxing its independence assumption, such as TAN.

TAN constructs the tree structure by finding a maximum weighted spanning tree [21] (see Figure 1b). The structure is determined by extending Chow-Liu tree [22], which uses CMI to measure the weight of arcs. The CMI between $X_{i}$ and $X_{j}$ given the class *Y*, $I(X_{i};X_{j}|Y)$, is defined as follows [23],

(7) $$I\left(X_{i};X_{j}|Y\right)=\sum_{x_{i}}\sum_{x_{j}}\sum_{y}P\left(x_{i},x_{j},y\right)\log\frac{P(x_{i},x_{j}|y)}{P\left(x_{i}|y\right)P\left(x_{j}|y\right)}$$

For each attribute $X_{i}\in X$, its parent set is $\pi_{i}=\left\{X_{j}\in X\right|$ $X_{j}\to X_{i}\in V\}$. The learning procedure of TAN is shown in Algorithm 1.
**Algorithm 1:** The Tree-augment Naive Bayes.
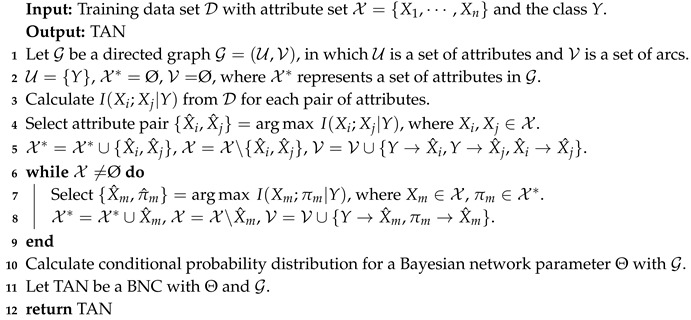


**Figure 2:**
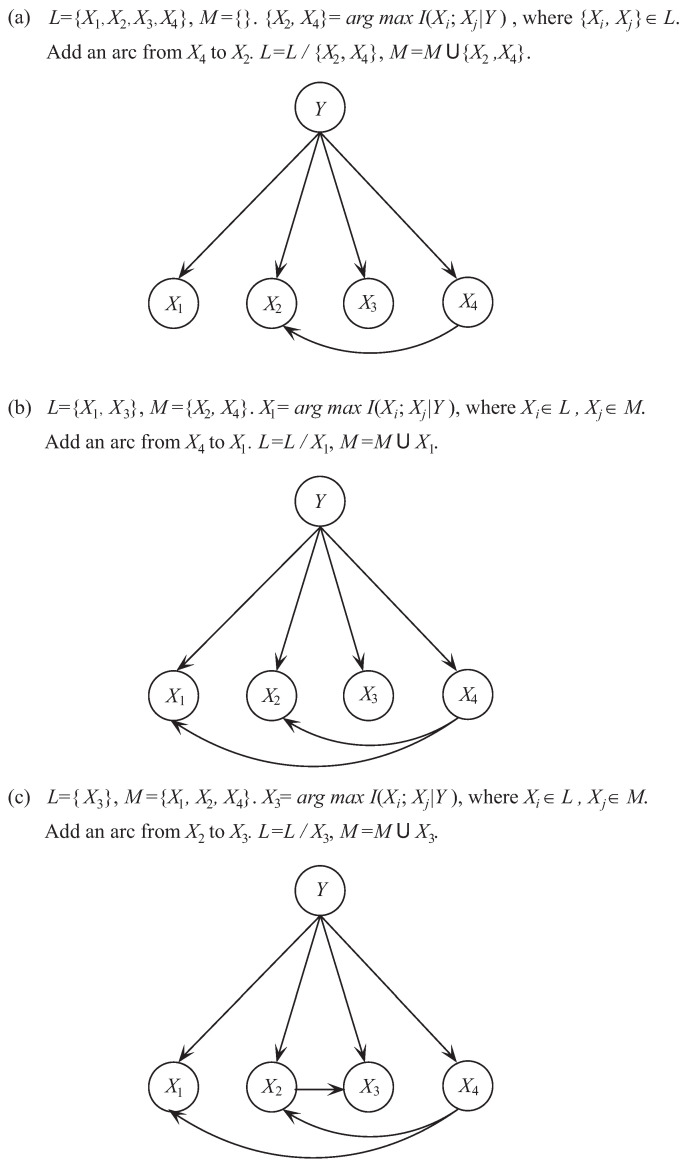
The learning procedure of TAN on Balance-Scale.

To illustrate the learning process of TAN, we take dataset Balance-Scale as an example. Dataset Balance-Scale is from the University of California Irvine (UCI) machine learning repository and has 625 instances, 4 attributes, and 3 class labels. As a 1-dependence BNC, TAN requires that each attribute can have at most 1 parent. In the first step we need to find the most significant dependence between attributes. As shown in Figure 2a, $I(X_{2};X_{4}|Y)$ corresponds to the maximum of $I(X_{i};X_{j}|Y)$ for any attribute pairs. Then arc $X_{2}-X_{4}$ is added to the topology of TAN. In the second step, we need to find the next significant dependence relationship between attributes. As shown in Figure 2b, $I(X_{1};X_{4}|Y)$ corresponds to the maximum of $I(X_{i};\Pi_{i}|Y)$ where $\Pi_{i}\in \left\{X_{2},X_{4}\right\}$ and $X_{i}\notin \left\{X_{2},X_{4}\right\}$. Then arc $X_{1}-X_{4}$ is added to the topology. The next iteration begins. As shown in Figure 2c, $I(X_{2};X_{3}|Y)$ corresponds to the maximum of $I(X_{i};\Pi_{i}|Y)$ where $\Pi_{i}\in \left\{X_{1},X_{2},X_{4}\right\}$ and $X_{i}\notin \left\{X_{1},X_{2},X_{4}\right\}$. Then arc $X_{2}-X_{3}$ is added to the topology. Finally, there exist at least 1 dependence relationship between any attribute $X_{i}$ and other attributes and the learning procedure of TAN stops.

According to the structure of TAN, $P_{TAN}\left(u\right)$ is calculated by

(8) $$P_{TAN}\left(u\right)=P\left(y\right)P\left(x_{1}|y\right)P\left(x_{2}|x_{1},y\right)\cdots P\left(x_{n}|\pi_{n},y\right)=P\left(y\right)P\left(x_{1}|y\right)\prod_{i=2}^{n}P\left(x_{i}|\pi_{i},y\right),$$

where $\pi_{i}$ (i>1) represents the parent attribute of $X_{i}$ (i>1). Correspondingly, $H_{TAN}\left(U\right)$ can be calculated by

(9) $$\begin{array}{cc} H_{TAN}\left(U\right) & =-\sum_{y,x_{1},\cdots ,x_{n}}P\left(y,x_{1},\cdots ,x_{n}\right)logP\left(y\right)P\left(x_{1}|y\right)P\left(x_{2}|x_{1},y\right)\cdots P\left(x_{n}|\pi_{n},y\right)\\ & =-\sum_{y,x_{1},\cdots ,x_{n}}P\left(y,x_{1},\cdots ,x_{n}\right)logP\left(y\right)-\sum_{y,x_{1},\cdots ,x_{n}}P\left(y,x_{1},\cdots ,x_{n}\right)logP\left(x_{1}|y\right)\\ & \;-\sum_{y,x_{1},\cdots ,x_{n}}\left\{P\left(y,x_{1},\cdots ,x_{n}\right)\sum_{i=2}^{n}logP\left(x_{i}|\pi_{i},y\right)\right\}\\ & =-\sum_{y}P\left(y\right)logP\left(y\right)-\sum_{y,x_{1}}P\left(y,x_{1}\right)logP\left(x_{1}|y\right)-\sum_{y,x_{i},\pi_{i}}\left\{P\left(y,x_{i},\pi_{i}\right)\sum_{i=2}^{n}logP\left(x_{i}|\pi_{i},y\right)\right\}\\ & =H\left(Y\right)+H\left(X_{1}|Y\right)+\sum_{i=2}^{n}H\left(X_{i}|\pi_{i},Y\right), \end{array}$$

According to Equation (6) and Equation (9), the difference between $H_{NB}\left(U\right)$ and $H_{TAN}\left(U\right)$ can be calculated by

(10) $$\begin{array}{cc} H_{NB}\left(U\right)-H_{TAN}\left(U\right) & =\left\{H\left(Y\right)+\sum_{i=1}^{n}H\left(X_{i}|Y\right)\right\}-\left\{H\left(Y\right)+H\left(X_{1}|Y\right)+\sum_{i=2}^{n}H\left(X_{i}|\pi_{i},Y\right)\right\}\\ & =\sum_{i=2}^{n}H\left(X_{i}|Y\right)-\sum_{i=2}^{n}H\left(X_{i}|\pi_{i},Y\right)\\ & =\sum_{i=2}^{n}I\left(X_{i};\pi_{i}|Y\right), \end{array}$$

Thus, the difference between $H_{NB}\left(U\right)$ and $H_{TAN}\left(U\right)$ is the summation of 1-order CMIs that correspond to the conditional dependence relationships among attributes. Equation (10) can clarify why TAN applies CMI to fully describe the 1-dependence relationships in the maximum weighted spanning tree. As TAN is a successful structure augmentation of NB, many researchers suggest that identifying significant dependencies can help to achieve more precise classification accuracy [24,25]. Ziebart et al. [26] model the selective forest-augmented naive Bayes by allowing attributes to be optionally dependent on the class. Jing and Pavlovi [27] presented the boosted BNC which greedily builds the structure with the arcs with the highest value of CMI.

KDB [28] extends the network structure further by using variable *k* to control the attribute dependence spectrum (see Figure 1c). KDB first sorts attributes by comparing MI $I(X_{i};Y)$. Suppose that the attribute order is $\left\{X_{1},X_{2},\cdots ,X_{n}\right\}$, $P_{KDB}\left(u\right)$ is calculated by

(11) $$\begin{array}{cc} P_{KDB}\left(u\right) & =P\left(y\right)P\left(x_{1}|y\right)P\left(x_{2}|x_{1},y\right)\cdots P\left(x_{k}|x_{1},\cdots ,x_{k-1},y\right)P\left(x_{k+1}|\Pi_{k+1},y\right)\cdots P\left(x_{n}|\Pi_{n},y\right)\\ & =P\left(y\right)P\left(x_{1}|y\right)\prod_{i=2}^{k}P\left(x_{i}|x_{1},\cdots ,x_{i-1},y\right)\prod_{j=k+1}^{n}P\left(x_{j}|\Pi_{j},y\right) \end{array}$$

where $\Pi_{i}$ is the set of *k* parent attributes of $X_{i}$ when k<i≤n. Whereas when k≥i, $X_{i}$ takes the first i−1 attributes in the order as its parent attributes. Correspondingly, $H_{KDB}\left(U\right)$ can be calculated by

(12) $$\begin{array}{cc} H_{KDB}\left(U\right) & =-\sum_{y,x_{1},\cdots ,x_{n}}P\left(y,x_{1},\cdots ,x_{n}\right)logP\left(y\right)P\left(x_{1}|y\right)P\left(x_{2}|x_{1},y\right)\cdots P\left(x_{n}|\Pi_{n},y\right)\\ & =-\sum_{y,x_{1},\cdots ,x_{n}}P\left(y,x_{1},\cdots ,x_{n}\right)logP\left(y\right)-\sum_{y,x_{1},\cdots ,x_{n}}P\left(y,x_{1},\cdots ,x_{n}\right)logP\left(x_{1}|y\right)\\ & \;-\sum_{y,x_{1},\cdots ,x_{n}}\sum_{i=2}^{k}P\left(y,x_{1},\cdots ,x_{n}\right)logP\left(x_{i}|x_{1},\cdots ,x_{i-1},y\right)\\ & \;-\sum_{y,x_{1},\cdots ,x_{n}}\sum_{j=k+1}^{n}P\left(y,x_{1},\cdots ,x_{n}\right)logP\left(x_{j}|\Pi_{j},y\right)\\ & =-\sum_{y}P\left(y\right)logP\left(y\right)-\sum_{y,x_{1}}P\left(y,x_{1}\right)logP\left(x_{1}|y\right)-\sum_{i=2}^{k}\sum_{y,x_{1},\cdots ,x_{i}}P\left(y,x_{1},\cdots ,x_{i}\right)logP\left(x_{i}|x_{1},\cdots ,x_{i-1},y\right)\\ & \;-\sum_{j=k+1}^{n}\sum_{y,x_{j},\Pi_{j}}P\left(y,x_{j},\Pi_{j}\right)logP\left(x_{j}|\Pi_{j},y\right)\\ & =H\left(Y\right)+H\left(X_{1}|Y\right)+\sum_{i=2}^{k}H\left(X_{i}|X_{1},\cdots ,X_{i-1},Y\right)+\sum_{j=k+1}^{n}H\left(X_{j}|\Pi_{j},Y\right) \end{array}$$

According to Equation (6) and Equation (12), the difference between $H_{NB}\left(U\right)$ and $H_{KDB}\left(U\right)$ can be calculated by

(13) $$\begin{array}{cc} & H_{NB}\left(U\right)-H_{KDB}\left(U\right)\\ = & \left\{H\left(Y\right)+\sum_{i=1}^{n}H\left(X_{i}|Y\right)\right\}-\left\{H\left(Y\right)+H\left(X_{1}|Y\right)+\sum_{i=2}^{k}H\left(X_{i}|X_{1},\cdots ,X_{i-1},Y\right)+\sum_{j=k+1}^{n}H\left(X_{j}|\Pi_{j},Y\right)\right\}\\ = & \sum_{i=2}^{k}I\left(X_{i};X_{1},\cdots ,X_{i-1}|Y\right)+\sum_{j=k+1}^{n}I\left(X_{j};\Pi_{j}|Y\right) \end{array}$$

Thus, the difference between $H_{NB}\left(U\right)$ and $H_{KDB}\left(U\right)$ is actually the summation of these CMIs of different orders.

Extending the network structure with high attribute dependence spectrum has become popular to improve the classification performance of BNCs [29]. Pernkopf and Bilmes [30] establish *k*-graphs via ranking attributes by means of a greedy algorithm and selecting the *k* best parents by scoring each possibility with the classification accuracy. Lu and Mineichi [31] propose *k*-dependence classifier chains with label-specific features and demonstrate the effectiveness of the method.

## 3. Extensive Tree-Augmented Naive Bayes

**Figure 3:**
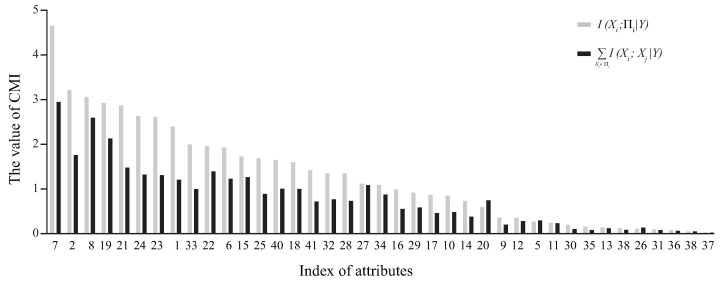
Distributions of $I(X_{i};\Pi_{i}|Y)$ and $\sum_{X_{j}\in \Pi_{i}}I\left(X_{i};X_{j}|Y\right)$ on Census-income.

KDB allows us to construct classifiers at arbitrary points (values of *k*) along the attribute dependence spectrum. To build an ideal KDB, we need to learn how to maximize the Kullback–Leibler divergence shown in Equation (13). The original KDB sort attributes by comparing MI and uses a set of 1-order CMIs (e.g., $I\left(X_{i};X_{1}|Y\right),I\left(X_{i};X_{2}|Y\right)\cdots ,I\left(X_{i};X_{i-1}|Y\right)$) rather than one higher-order CMI (e.g., $I(X_{i};X_{1},\cdots ,X_{i-1}|Y)$) to measure the conditional dependencies between $X_{i}$ and its parent attributes. To illustrate the difference between these two measures, we take data set Census-income, which has 299,285 instances, 41 attributes and 2 class labels, for example to learn specific KDB, corresponding distributions of $\sum_{X_{j}\in \Pi_{i}}I\left(X_{i};X_{j}|Y\right)$ and $I(X_{i};\Pi_{i}|Y)$ are shown in Figure 3, where the X-axis denotes the index of attributes sorted in the decreasing order of $I(X_{i};\Pi_{i}|Y)$. From Figure 3, the distribution of $\sum_{X_{j}\in \Pi_{i}}I\left(X_{i};X_{j}|Y\right)$ differs greatly to that of $I(X_{i};\Pi_{i}|Y)$, thus the former is not appropriate to approximate the latter.

CMI or $I(X_{i};X_{j}|Y)$ is a popular measure to evaluate the dependency relationship between attributes, and the maximum weighted spanning tree learned by TAN describes the most significant dependencies in its 1-dependence structure. While $I(X_{i};X_{j}|Y)$ may fail to discriminate the dependency relationships given different class labels. The definition of CMI shown in Equation (7) can be represented as follows,

(14) $$\begin{array}{cc} I(X_{i};X_{j}|Y) & =\sum_{x_{i}}\sum_{x_{j}}\sum_{y}P\left(x_{i},x_{j},y\right)\log\frac{P(x_{i},x_{j}|y)}{P\left(x_{i}|y\right)P\left(x_{j}|y\right)}\\ & =\sum_{y}P\left(y\right)\left\{\sum_{x_{i}}\sum_{x_{j}}P\left(x_{i},x_{j}|y\right)\log\frac{P(x_{i},x_{j}|y)}{P\left(x_{i}|y\right)P\left(x_{j}|y\right)}\right\}\\ & =\sum_{y}P\left(y\right)I\left(X_{i};X_{j}|y\right) \end{array}$$

where $I(X_{i};X_{j}|y)$ measures the informational correlation between $X_{i}$ and $X_{j}$ given specific class label *y* and is defined as follows,

(15) $$I\left(X_{i};X_{j}|y\right)=\sum_{x_{i}}\sum_{x_{j}}P\left(x_{i},x_{j}|y\right)\log\frac{P(x_{i},x_{j}|y)}{P\left(x_{i}|y\right)P\left(x_{j}|y\right)},$$

**Figure 4:**
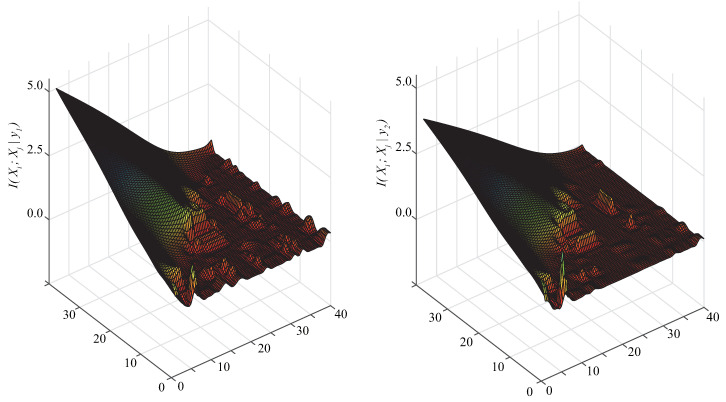
$I(X_{i};X_{j}|y)$ with different class labels on Census-income.

From Equation (1), for restricted BNCs the most important issue is how to deeply mine the significant conditional dependencies among attributes given class label *y*. Whereas from Equation (14), CMI or $I(X_{i};X_{j}|Y)$ is just a simple uniform averaging of $I(X_{i};X_{j}|y)$, and the latter assumes that the data set has been divided into |Y| subsets and each subset corresponds a specific class label. To illustrate the variety of $I(X_{i};X_{j}|y)$ with different class labels, we present their comparison on data set Census-income in Figure 4. As Figure 4 shows, the distribution of $I(X_{i};X_{j}|y)$ differs greatly as *y* changes. Correspondingly the network structures, in which the conditional dependencies are measured by $I(X_{i};X_{j}|y)$, should be different.

According to Figure 3 and Figure 4, $\sum_{X_{j}\in \Pi_{i}}I\left(X_{i};X_{j}|Y\right)$ may not be able to approximate $I(X_{i};\Pi_{i}|Y)$, and to discriminate the conditional dependence between attributes, $I(X_{i},X_{j}|y)$ rather than $I(X_{i},X_{j}|Y)$ is appropriate to measure the conditional dependencies implicated in different subspaces of training data. Thus, motivated by the learning schemes of TAN and KDB, the structure of the proposed BNC is just like an extension of TAN from 1-dependence BNC to arbitrary *k*-dependence BNC, and we respectively learn |Y| sub-classifiers from |Y| subspaces of training data. The attributes are sorted in such a way that the Kullback–Leibler divergence will be maximized and each attribute can have at most *k* parent attributes. Supposed that the attribute order is $\left\{X_{1},X_{2},\cdots ,X_{n}\right\}$, from the chain rule of joint probability of BNC (see Equation (2)) we know that any possible parents of attribute $X_{i}$ must be selected from $\left\{X_{1},X_{2},\cdots ,X_{i-1}\right\}$. As a *k*-dependence BNC, ETAN uses a heuristic search strategy and the learning procedure of ETAN is divided into two parts: when i≤k+1, all attributes in $\left\{X_{1},X_{2},\cdots ,X_{i-1}\right\}$ will be selected as the parents of $X_{i}$; when i>k+1, only *k* attributes in $\left\{X_{1},X_{2},\cdots ,X_{i-1}\right\}$ will be selected as the parents of $X_{i}$. The learning procedure of one sub-model of ETAN is shown in Algorithm 2.
**Algorithm 2:** Sub-ETAN (*y*,*k*).
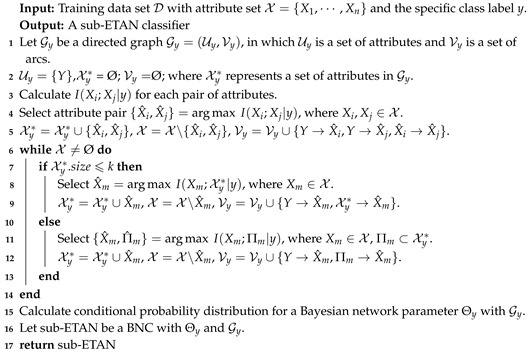


**Figure 5:**
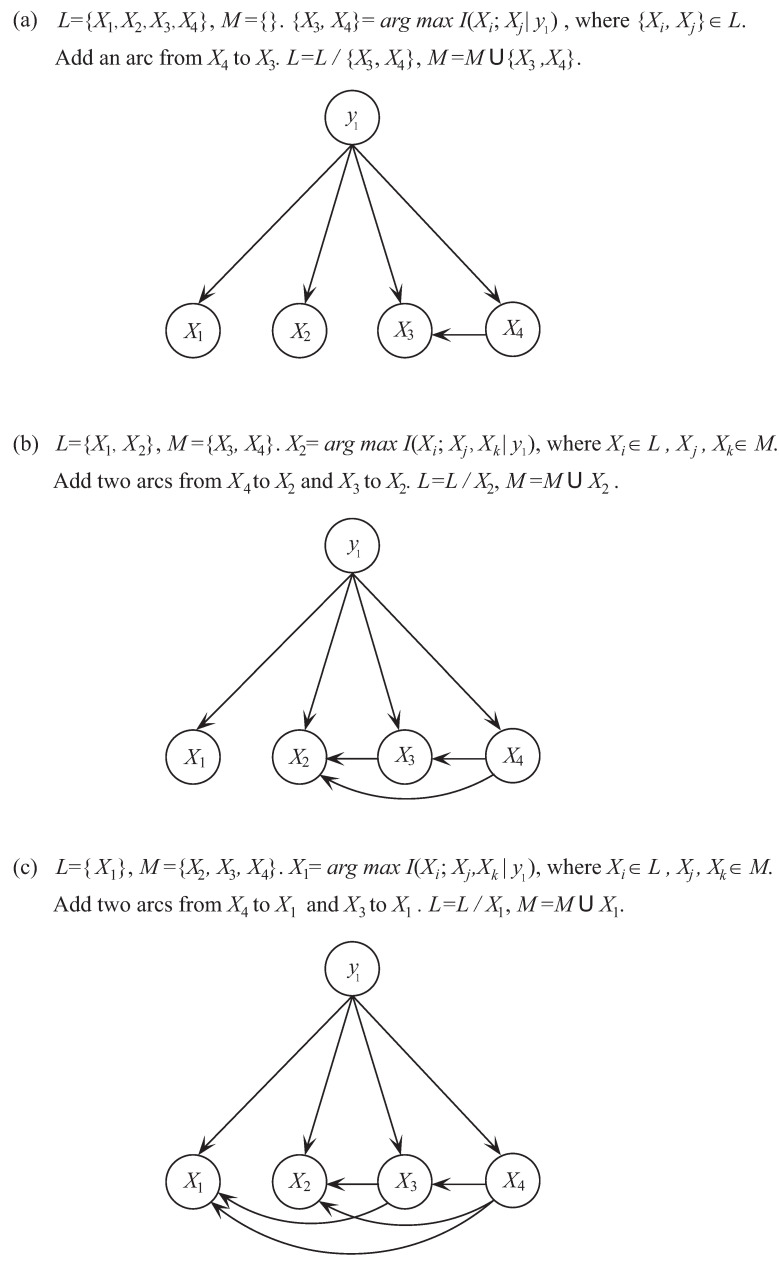
The learning procedure of ETAN with *k* = 2 on Balance-Scale.

To illustrate the learning process of ETAN, we also take dataset Balance-Scale as an example and set $Y=y_{1}$, k=2. In the first step we need to find the most significant dependence between attributes. As shown in Figure 5a, $I(X_{3};X_{4}|y_{1})$ corresponds to the maximum of $I(X_{i};X_{j}|y_{1})$ for any attribute pairs. Then arc $X_{3}-X_{4}$ is added to the topology of ETAN. In the second step, we need to find the next significant dependence relationship between attributes. As shown in Figure 5b, $I(X_{2};X_{3},X_{4}|y_{1})$ corresponds to the maximum of $I(X_{i};\Pi_{i}|y_{1})$ where $\Pi_{i}\in \left\{X_{3},X_{4}\right\}$ and $X_{i}\notin \left\{X_{3},X_{4}\right\}$. Then arcs $X_{2}-X_{4}$ and $X_{2}-X_{3}$ are added to the topology. The next iteration begins. As shown in Figure 5c, $I(X_{1};X_{3},X_{4}|y_{1})$ corresponds to the maximum of $I(X_{i};\Pi_{i}|y_{1})$ where $\Pi_{i}\in \left\{X_{2},X_{3},X_{4}\right\}$ and $X_{i}\notin \left\{X_{2},X_{3},X_{4}\right\}$. Then arcs $X_{1}-X_{3}$ and $X_{1}-X_{4}$ are added to the topology. Finally, there exist at least *k* dependence relationship between any attribute $X_{i}$ and other attributes and the learning procedure of ETAN stops.

For testing instance x, its class label *y* will correspond to one of the |Y| candidate classifiers, whose structure may lead to the maximum of joint probability P(y,x), i.e., $y^{*}=\arg\max P\left(y,x\right|$BNC). The prediction procedure of our proposed method, the extensive tree-augmented naive Bayes, is shown in Algorithm 3.
**Algorithm 3:** The Extensive tree-augmented naive Bayes (ETAN).
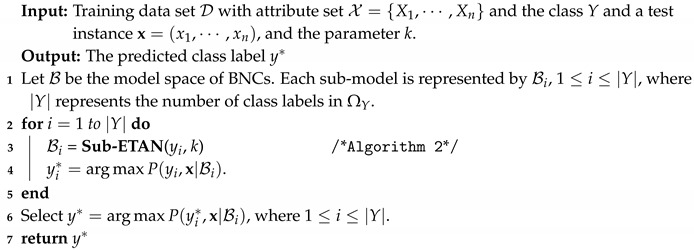


## 4. Experimental Results

We compare the performance of our proposed methods with other algorithms. All experiments are carried out on 40 data sets from UCI machine learning repository. Table 1 shows the details of each data set used, including the number of instances, attributes, and the class. These data sets are arranged in the order of the number of instances. For each data set, numeric attributes are discretized using Minimum Description Length discretization [32]. To allow the proposed algorithm to be compared with Weka’s algorithms, missing values for qualitative attributes are replaced with modes and those for quantitative attributes are replaced with means from the training data.

The following algorithms are compared for experimental study,

- NB, naive Bayes.
- TAN, standard tree-augmented naive Bayes.
- KDB, *k*-dependence Bayesian classifier with k=2.
- LR, Logistic Regression.
- ETAN, Extensive TAN with k=2.
- AODE, averaged one-dependence estimators [33].
- WAODE, weighted averaged one-dependence estimators [34].

In machine learning, zero-one loss is the most common function to measure the classification performance. Kohavi and Wolpert [35] presented a bias-variance decomposition of zero-one loss for analyzing supervised learning scenarios. Bias represents the systematic component of error, which measures how closely the classifier can describe surfaces for a data set. Variance represents the component of error that stems from sampling, which represents the sensitivity of the classifier to changes in training data. The estimation of these measures is using 10-fold cross validation to provide an accurate evaluation of the performance of algorithms. The experimental results of zero-one loss, bias and variance are shown in Table A1–Table A3 respectively in Appendix A. Statistically, we employ win/draw/loss when two algorithms are compared with respect to a performance measure. A win and a draw respectively indicate that the algorithm has significantly and not significantly lower error than the comparator. We assess a difference as significant if the outcome of a one-tailed binomial sign test is less than 0.05. Base probability estimates with *M*-estimation which leads to more accurate probabilities are applied in our paper, where the value of *M* is 1 [36].

### 4.1. ETAN vs. BNCs

In this section, we present experimental results of our proposed algorithms, ETAN. Table 2 displays the win/draw/loss records summarizing the relative zero-one loss, bias, and variance of different algorithms. Cell [i,j] in Table 2 contains win/draw/loss records for the BNC on row *i* against the BNC on column *j*.

As shown in Table 2, ETAN respectively performs significantly better than NB on 28 datasets in terms of zero-one loss. In particular, ETAN shows obvious advantages when compared with TAN (19 wins). ETAN has a clear edge over KDB with win/draw/loss records of 17/14/9. Ensemble of classifiers, e.g., AODE and WAODE, brings improvement in accuracy in the sense that small variations in the training set would lead them to produce very different models, which help achieve higher classification accuracy compared to these single-structure classifiers. ETAN is also an ensemble but has higher attribute dependence spectrum than AODE or WAODE. It retains the advantage over AODE and WAODE in terms of zero-one loss, although less significantly. As structure complexity increases, ETAN uses higher-order CMI to measure the high-dependence relationships that may help to improve the classification performance.

The win/draw/loss records of bias and variance are also shown in Table 2. Bias-variance analysis of BNCs is given in the following discussion. By modeling BNCs with respect to the class labels, ETAN makes full use of the training data. For bias, ETAN performs better than NB (32/4/4). When ETAN is compared with 1-dependence classifier, ETAN beats TAN on 17 datasets and loses on 8 datasets. As each sub-model in ETAN is *k*-dependence classifier, ETAN beats AODE on 20 datasets. It indicates that BNCs with more interdependencies can perform better in terms of bias. Variance-wise, NB performs the best, because the structure of NB is definite and insensitive to variations in training data. For single-structure BNCs, higher-dependence BNCs (e.g., KDB) performs worse than lower-dependence BNCs (e.g., TAN). This also holds for ensemble classifiers, and ETAN performs worse than AODE and WAODE. The reason may be that further dependence discovery will result in overfitting.

ETAN is a structural augmentation of TAN where every attribute takes the class variable and at most *k* other attributes as its parents. *k*-order CMI is introduced to measure the conditional dependencies among attributes and the final structure is an extended maximum spanning tree. This alleviates some of NB’s independence assumption and therefore reduces its bias at the expense of increasing its variance. As can be seen from Table 2, ETAN performs better in terms of variance than NB on 4 datasets, i.e., Anneal, Vowel, Dis and Mushrooms. Each dataset has smaller number of instances and larger number of attributes. That may lead to sparsely distributed data and imprecise estimate of probability distribution. For lower quantities of data, the lower variance results in lower error for ETAN, while for larger quantities of data the lower bias results in lower error. ETAN may underfit the sparsely distributed training data that will lead to lower variance and then higher classification accuracy. The ideal datasets on which ETAN has better variance prediction accuracy are those with small data quantity and sparse data. For example, dataset Anneal has only 898 instances but 38 attributes and 6 class labels.

### 4.2. ETAN vs. Logistic Regression (LR)

In this section, our proposed algorithms are compared with the state-of-the-art algorithm, Logistic Regression (LR). LR can be viewed as a partially parametric approach [37], hence, a BNC can be mapped to a LR model [38]. We use LR’s implementation in Weka, an open source provided by the University of Waikato for machine learning. Weka offers an improved implementation of LR, which use a quasi-Newton to search for the optimized values of attributes and considers the instance weights. The experimental results in terms of zero-one one loss, bias and variance have been shown in the fifth column of Table A1–Table A3 in Appendix A. Table 3 shows the win/draw/loss results. Due to the computational constraints of LR, the size of data sets has an obvious effect on its training time; hence, we have not been able to learn the classification models for the two largest data sets. That is why the sum of the number of all cells is exactly 38.

As we can see from Table 3, ETAN beats LR on 26 data sets, which means ETAN achieves better classification performance than LR. ETAN results in not only better bias performance on 22 data sets but also better variance performance on 21 data sets. In the other words, ETAN is difficult to be beaten by LR.

**Figure 6:**
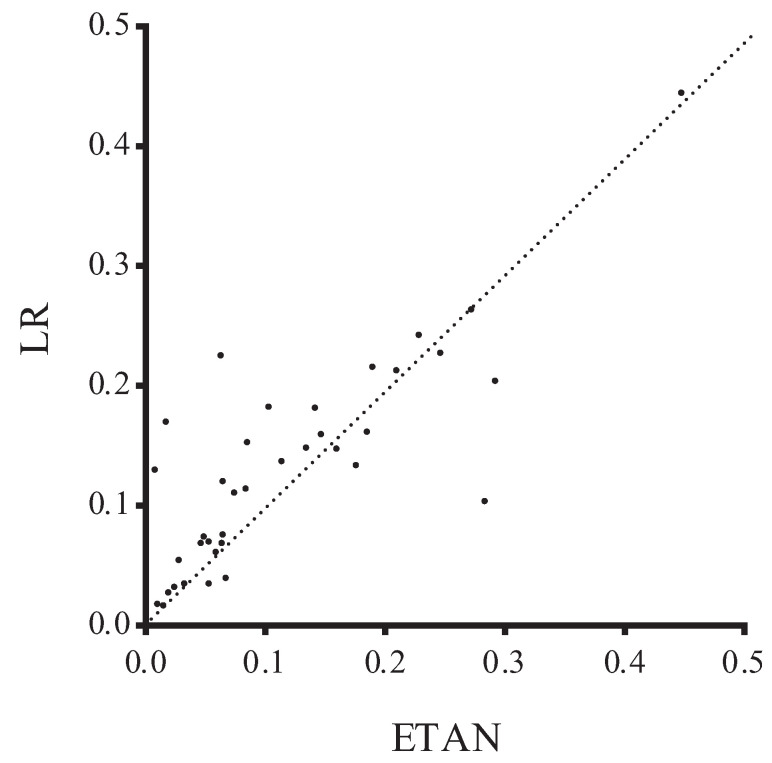
Comparison between LR and ETAN in terms of zero-one loss.

To further illustrate the advantages of our algorithms, we present the comparison results with respect to zero-one loss in Figure 6, where the *X*-axis represents the zero-one loss results of ETAN and the *Y*-axis represents those of LR. Most of the points in Figure 6 are above the diagonal lines, which means that our algorithms have shown better classification performance in general. LR is a popular binary classifier and attempts to predict outcomes based on a set of independent attributes. Among those data sets which correspond to the points under the diagonal lines, most of them have 2 class labels, less than 1000 instances and at least 8 attributes. Obviously, the sparsely distributed data and binary classification may be the main reasons why LR performs better. However, for data sets containing non-binary attributes, ETAN allows us to build (more expressive) non-linear classifiers, which is impossible for LR, unless one “binarizes” all attributes and this may artificially introduce noise.

### 4.3. Comparison of All Algorithms

To compare multiple algorithms over multiple data sets, Friedman test is used in the following discussion, which ranks the algorithms for each data set [39]. We calculate the rank of each algorithm for each data set separately (assign average ranks in case of tie). The null hypothesis is that there is no significant difference in the average ranks. The Friedman statistic is distributed according to $\chi_{F}^{2}$ with t−1 degrees of freedom. For any level of significance α, the null hypothesis will be rejected if $\chi_{F}^{2}>\chi_{\alpha }^{2}$. The critical value of $\chi_{\alpha }^{2}$ for α= 0.05 with t−1= 6 degrees of freedom is 14.07. The Friedman statistic for zero-one loss is 37.90. Therefore, the null hypothesis is rejected.

As the null hypothesis is rejected, we perform Nemenyi test which is used to analyze which pairs of algorithms are significantly different [40]. If the difference between the average ranks of two algorithms is less than the critical difference (CD), their performance is significantly different. For these 7 algorithms and 38 data sets, the value of CD is 1.462.

**Figure 7:**
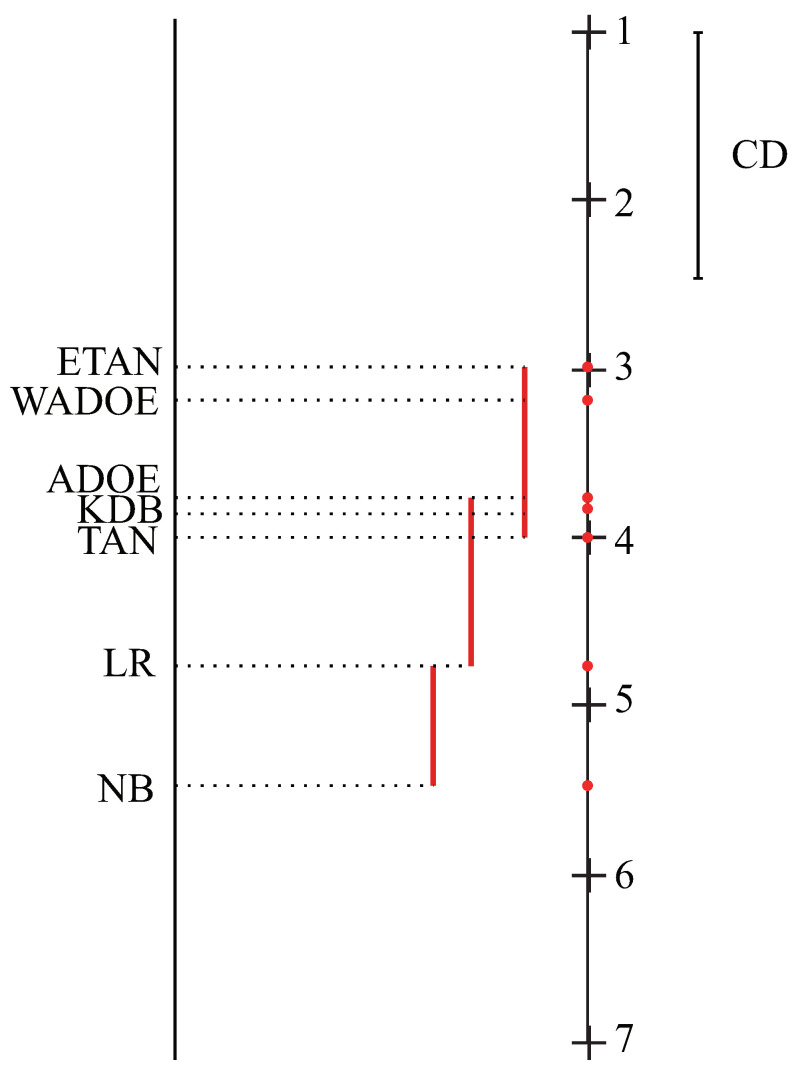
Nemenyi test for all algorithms.

The comparison of all algorithms against each other with Nemenyi test in terms of zero-one loss is shown in Figure 7. We plot the algorithms on the left line according to average ranks, the higher the position of algorithms, the lower average ranks will be, hence the better performance. As we can see, the rank of ETAN is significantly better than that of other algorithms. WAODE and AODE also achieve lower average rank than KDB, TAN, and NB. It indicates that ensemble classifiers may help to improve performance of the single-structure classifiers. The advantage of ETAN over ADOE and that of KDB over TAN may be attributed to the increasing attribute dependence spectrum.

## 5. Conclusions

Our work was primarily motivated by the consideration that the structure difference between NB and its variations can be measured by different orders of CMIs in terms of Kullback–Leibler divergence, and conditional dependencies between attributes may vary greatly for different class labels. In this paper, we provide a novel learning algorithm, ETAN, which extends TAN to arbitrary *k*-dependence BNC. The final network structure is similar to an extended version of maximum weighted spanning tree and corresponds to the maximum of sum of CMIs. ETAN substantially achieves better performance with respect to different evaluation functions and is highly competitive with the state-of-the-art higher-dependence BNC, e.g., KDB.

## Figures and Tables

**Table 1:** Data sets.

No.	Data Set	Ins.	Att.	Class	No.	Data Set	Inst.	Att.	Class
1	Labor	57	16	2	21	Vowel	990	13	11
2	Labor-Negotiations	57	16	2	22	Led	1000	7	10
3	Lymphography	150	4	3	23	Car	1728	6	4
4	Iris	150	4	3	24	Hypothyroid	3163	25	2
5	Hungarian	294	13	2	25	Dis	3772	29	2
6	Heart-Disease-C	303	13	2	26	Sick	3772	29	2
7	Soybean-Large	307	35	19	27	Abalone	4177	8	3
8	Ionosphere	351	34	2	28	Spambase	4601	57	2
9	House-Votes-84	435	16	2	29	Waveform-5000	5000	40	3
10	Musk1	476	166	2	30	Page-Blocks	5473	10	5
11	Cylinder-Bands	540	39	2	31	Optdigits	5620	64	10
12	Chess	551	39	2	32	Satellite	6435	36	6
13	Syncon	600	60	6	33	Mushrooms	8124	22	2
14	Balance-Scale	625	4	3	34	Thyroid	9169	29	20
15	Soybean	683	35	19	35	Letter-Recog	20000	26	2
16	Credit-A	690	15	2	36	Adult	48842	14	2
17	Breast-Cancer-W	699	9	2	37	Connect-4	67557	42	3
18	Pima-Ind-Diabetes	768	8	2	38	Waveform	100000	21	3
19	Vehicle	846	18	4	39	Census-Income	299285	41	2
20	Anneal	898	38	6	40	Poker-Hand	1025010	10	10

**Table 2:** The records of win/draw/loss for BNCs and our algorithms.

	BNC	NB	TAN	KDB	AODE	WAODE
	TAN	27/5/8	-	-	-	-
	KDB	25/10/5	16/13/11	-	-	-
Zero-one loss	AODE	29/8/3	13/15/12	13/14/13	-	-
	WAODE	28/7/5	19/14/7	18/13/9	14/19/7	-
	ETAN	30/6/4	21/11/8	19/12/9	18/13/9	15/15/10
	TAN	28/5/7	-	-	-	-
	KDB	26/8/6	18/14/8	-	-	-
Bias	AODE	31/7/2	14/10/16	13/6/21	-	-
	WAODE	24/2/14	19/4/17	18/4/18	18/4/18	-
	ETAN	32/4/4	18/14/8	9/19/12	20/13/7	19/3/18
	TAN	6/2/32	-	-	-	-
	KDB	9/2/29	10/7/23	-	-	-
Variance	AODE	10/11/19	30/3/7	29/3/8	-	-
	WAODE	12/5/22	21/3/16	21/1/18	12/4/24	-
	ETAN	4/5/31	15/8/17	24/6/10	3/6/31	18/3/19

**Table 3:** The records of win/draw/loss for LR and our algorithms.

		LR
	Zero-one loss	26/4/8
ETAN	Bias	22/5/11
	Variance	21/3/14

## Appendix A. Detailed Experimental Results

**Table A1:** Experimental results of zero-one loss.

Data Set	NB	TAN	KDB	LR	ETAN	AODE	WAODE
Labor	0.0289	0.0211	0.0279	0.0211	0.0274	0.0205	0.0200
Labor-Negotiations	0.0505	0.0763	0.0553	0.0422	0.0237	0.0268	0.0268
Lymphography	0.0902	0.0976	0.1041	0.1422	0.0814	0.0853	0.0951
Iris	0.0590	0.0550	0.0656	0.0343	0.0760	0.0626	0.0624
Hungarian	0.1646	0.1454	0.1480	0.1057	0.1456	0.1597	0.1611
Heart-Disease-C	0.1297	0.1263	0.1299	0.1300	0.1300	0.1171	0.1092
Soybean-Large	0.1070	0.1275	0.1086	0.1104	0.0964	0.0812	0.0655
Ionosphere	0.1220	0.0800	0.0855	0.1117	0.0817	0.0903	0.0061
House-Votes-84	0.0899	0.0410	0.0258	0.0307	0.0406	0.0518	0.0406
Musk1	0.1847	0.1563	0.1535	0.1357	0.1527	0.1670	0.1501
Cylinder-Bands	0.2000	0.3242	0.1939	0.1863	0.1746	0.1684	0.1286
Chess	0.1413	0.1427	0.1119	0.0832	0.1192	0.1380	0.0180
Syncon	0.0516	0.0203	0.0314	0.1123	0.0339	0.0334	0.1827
Balance-Scale	0.1840	0.1843	0.1902	0.0753	0.1877	0.1905	0.0503
Soybean	0.1015	0.0504	0.0491	0.0656	0.0622	0.0690	0.0900
Credit-A	0.0912	0.1171	0.1137	0.1279	0.1266	0.0892	0.0953
Breast-Cancer-W	0.0187	0.0315	0.0449	0.0375	0.0263	0.0243	0.1941
Pima-Ind-Diabetes	0.1957	0.1946	0.1944	0.1898	0.1905	0.1935	0.2398
Vehicle	0.3330	0.2384	0.2494	0.1540	0.2482	0.2435	0.0194
Anneal	0.0354	0.0195	0.0073	0.0788	0.0186	0.0180	0.2104
Vowel	0.3301	0.1931	0.1745	0.1688	0.2135	0.2268	0.1811
Led	0.2322	0.2247	0.2317	0.2211	0.2348	0.2327	0.3766
Car	0.0937	0.0478	0.0387	0.0536	0.0524	0.0597	0.0633
Hypothyroid	0.0116	0.0105	0.0096	0.0181	0.0095	0.0094	0.0315
Dis	0.0165	0.0194	0.0191	0.0195	0.0194	0.0163	0.0078
Sick	0.0246	0.0208	0.0198	0.0277	0.0215	0.0221	0.3212
Abalone	0.4180	0.3134	0.3033	0.3613	0.3089	0.3201	0.0574
Spambase	0.0929	0.0571	0.0497	0.0560	0.0488	0.0631	0.1184
Waveform-5000	0.1762	0.1232	0.1157	0.1207	0.1122	0.1233	0.2172
Page-Blocks	0.0451	0.0306	0.0280	0.0282	0.0259	0.0259	0.0224
Optdigits	0.0685	0.0275	0.0250	0.0382	0.0182	0.0203	0.0902
Satellite	0.1746	0.0948	0.0808	0.1064	0.0834	0.0889	0.0002
Mushrooms	0.0237	0.0001	0.0001	0.0000	0.0001	0.0004	0.0561
Thyroid	0.0994	0.0572	0.0553	0.0999	0.0535	0.0658	0.0561
Letter-Recog	0.2207	0.1032	0.1387	0.1945	0.0569	0.0806	0.0892
Adult	0.1649	0.1312	0.1220	0.1394	0.1215	0.1440	0.0006
Connect-4	0.2660	0.2253	0.2022	0.2279	0.1981	0.2279	0.0158
Waveform	0.0219	0.0152	0.0210	0.0267	0.0140	0.0157	0.3068
Census-Income	0.2303	0.0544	0.0421	-	0.0450	0.0859	0.2083
Poker-Hand	0.4979	0.2865	0.1326	-	0.4040	0.4217	0.1716

**Table A2:** Experimental results of Bias.

Data Set	NB	TAN	KDB	LR	ETAN	AODE	WAODE
Labor	0.0289	0.0211	0.0279	0.0211	0.0274	0.0205	0.0200
Labor-Negotiations	0.0505	0.0763	0.0553	0.0422	0.0237	0.0268	0.0268
Lymphography	0.0902	0.0976	0.1041	0.1422	0.0814	0.0853	0.0951
Iris	0.0590	0.0550	0.0656	0.0343	0.0760	0.0626	0.0624
Hungarian	0.1646	0.1454	0.1480	0.1057	0.1456	0.1597	0.1611
Heart-Disease-C	0.1297	0.1263	0.1299	0.1300	0.1300	0.1171	0.1092
Soybean-Large	0.1070	0.1275	0.1086	0.1104	0.0964	0.0812	0.0655
Ionosphere	0.1220	0.0800	0.0855	0.1117	0.0817	0.0903	0.0061
House-Votes-84	0.0899	0.0410	0.0258	0.0307	0.0406	0.0518	0.0406
Musk1	0.1847	0.1563	0.1535	0.1357	0.1527	0.1670	0.1501
Cylinder-Bands	0.2000	0.3242	0.1939	0.1863	0.1746	0.1684	0.1286
Chess	0.1413	0.1427	0.1119	0.0832	0.1192	0.1380	0.0180
Syncon	0.0516	0.0203	0.0314	0.1123	0.0339	0.0334	0.1827
Balance-Scale	0.1840	0.1843	0.1902	0.0753	0.1877	0.1905	0.0503
Soybean	0.1015	0.0504	0.0491	0.0656	0.0622	0.0690	0.0900
Credit-A	0.0912	0.1171	0.1137	0.1279	0.1266	0.0892	0.0953
Breast-Cancer-W	0.0187	0.0315	0.0449	0.0375	0.0263	0.0243	0.1941
Pima-Ind-Diabetes	0.1957	0.1946	0.1944	0.1898	0.1905	0.1935	0.2398
Vehicle	0.3330	0.2384	0.2494	0.1540	0.2482	0.2435	0.0194
Anneal	0.0354	0.0195	0.0073	0.0788	0.0186	0.0180	0.2104
Vowel	0.3301	0.1931	0.1745	0.1688	0.2135	0.2268	0.1811
Led	0.2322	0.2247	0.2317	0.2211	0.2348	0.2327	0.3766
Car	0.0937	0.0478	0.0387	0.0536	0.0524	0.0597	0.0633
Hypothyroid	0.0116	0.0105	0.0096	0.0181	0.0095	0.0094	0.0315
Dis	0.0165	0.0194	0.0191	0.0195	0.0194	0.0163	0.0078
Sick	0.0246	0.0208	0.0198	0.0277	0.0215	0.0221	0.3212
Abalone	0.4180	0.3134	0.3033	0.3613	0.3089	0.3201	0.0574
Spambase	0.0929	0.0571	0.0497	0.0560	0.0488	0.0631	0.1184
Waveform-5000	0.1762	0.1232	0.1157	0.1207	0.1122	0.1233	0.2172
Page-Blocks	0.0451	0.0306	0.0280	0.0282	0.0259	0.0259	0.0224
Optdigits	0.0685	0.0275	0.0250	0.0382	0.0182	0.0203	0.0902
Satellite	0.1746	0.0948	0.0808	0.1064	0.0834	0.0889	0.0002
Mushrooms	0.0237	0.0001	0.0001	0.0000	0.0001	0.0004	0.0561
Thyroid	0.0994	0.0572	0.0553	0.0999	0.0535	0.0658	0.0561
Letter-Recog	0.2207	0.1032	0.1387	0.1945	0.0569	0.0806	0.0892
Adult	0.1649	0.1312	0.1220	0.1394	0.1215	0.1440	0.0006
Connect-4	0.2660	0.2253	0.2022	0.2279	0.1981	0.2279	0.0158
Waveform	0.0219	0.0152	0.0210	0.0267	0.0140	0.0157	0.3068
Census-Income	0.2303	0.0544	0.0421	-	0.0450	0.0859	0.2083
Poker-Hand	0.4979	0.2865	0.1326	-	0.4040	0.4217	0.1716

**Table A3:** Experimental results of Variance.

Data Set	NB	TAN	KDB	LR	ETAN	AODE	WAODE
Labor	0.0395	0.0632	0.0721	0.0328	0.0779	0.0268	0.0221
Labor-Negotiations	0.0653	0.1395	0.1289	0.0655	0.0868	0.0626	0.0626
Lymphography	0.0343	0.1106	0.1408	0.1212	0.0961	0.0412	0.0478
Iris	0.0390	0.0510	0.0364	0.0327	0.0460	0.0394	0.0396
Hungarian	0.0201	0.0556	0.0561	0.0751	0.0411	0.0270	0.0317
Heart-Disease-C	0.0248	0.0479	0.0582	0.0920	0.0591	0.0304	0.0383
Soybean-Large	0.0783	0.1127	0.0982	0.1542	0.0899	0.0747	0.0855
Ionosphere	0.0242	0.0414	0.0581	0.0946	0.0448	0.0319	0.0242
House-Votes-84	0.0066	0.0170	0.0197	0.0714	0.0083	0.0068	0.0123
Musk1	0.1108	0.1191	0.1320	0.1691	0.1157	0.1153	0.1010
Cylinder-Bands	0.0656	0.0724	0.0750	0.1437	0.0888	0.0827	0.0364
Chess	0.0401	0.0491	0.0531	0.0791	0.0578	0.0385	0.0230
Syncon	0.0204	0.0222	0.0301	0.1764	0.0246	0.0161	0.0913
Balance-Scale	0.0848	0.0941	0.0872	0.0339	0.0863	0.0854	0.0334
Soybean	0.0302	0.0593	0.0439	0.0839	0.0395	0.0288	0.0321
Credit-A	0.0249	0.0555	0.0768	0.0737	0.0673	0.0269	0.0264
Breast-Cancer-W	0.0010	0.0372	0.0504	0.0395	0.0376	0.0118	0.0700
Pima-Ind-Diabetes	0.0715	0.0663	0.0689	0.0425	0.0697	0.0729	0.1276
Vehicle	0.1120	0.1297	0.1283	0.0797	0.1330	0.1246	0.0161
Anneal	0.0168	0.0156	0.0152	0.0593	0.0139	0.0118	0.0604
Vowel	0.2542	0.2466	0.2325	0.2239	0.2310	0.2465	0.2489
Led	0.0333	0.0536	0.0565	0.0640	0.0460	0.0372	0.1106
Car	0.0520	0.0375	0.0434	0.0385	0.0427	0.0431	0.0509
Hypothyroid	0.0031	0.0029	0.0024	0.0062	0.0039	0.0026	0.0083
Dis	0.0069	0.0006	0.0011	0.0038	0.0009	0.0048	0.0056
Sick	0.0047	0.0052	0.0043	0.0084	0.0063	0.0038	0.1543
Abalone	0.0682	0.1690	0.1769	0.0746	0.1679	0.1536	0.0111
Spambase	0.0092	0.0157	0.0214	0.0243	0.0177	0.0098	0.0420
Waveform-5000	0.0259	0.0687	0.0843	0.0310	0.0693	0.0403	0.1311
Page-Blocks	0.0135	0.0144	0.0177	0.0123	0.0139	0.0111	0.0137
Optdigits	0.0153	0.0185	0.0254	0.0752	0.0162	0.0133	0.0364
Satellite	0.0139	0.0368	0.0455	0.0517	0.0395	0.0325	0.0001
Mushrooms	0.0043	0.0002	0.0002	0.0001	0.0001	0.0001	0.0239
Thyroid	0.0205	0.0252	0.0272	0.0453	0.0239	0.0202	0.0241
Letter-Recog	0.0471	0.0591	0.0113	0.0422	0.0523	0.0709	0.0417
Adult	0.0069	0.0165	0.0285	0.0108	0.0236	0.0104	0.0004
Connect-4	0.0156	0.0149	0.0309	0.0127	0.0373	0.0199	0.0023
Waveform	0.0009	0.0053	0.0037	0.0024	0.0059	0.0023	0.0632
Census-Income	0.0052	0.0100	0.0110	-	0.0144	0.0138	0.0224
Poker-Hand	0.0000	0.0424	0.0633	-	0.0440	0.0273	0.0602
